# Elevated S100A9 expression in chronic rhinosinusitis coincides with elevated MMP production and proliferation in vitro

**DOI:** 10.1038/s41598-020-73480-8

**Published:** 2020-10-01

**Authors:** Marina Boruk, Christopher Railwah, Alnardo Lora, Sridesh Nath, Derek Wu, Lillian Chow, Panid Borhanjoo, Abdoulaye J. Dabo, Sadakat Chowdhury, Ryan Kaiser, Robert F. Foronjy, Richard Rosenfeld, Patrick Geraghty

**Affiliations:** 1 Department of Otolaryngology, State University of New York Downstate Medical Center, Brooklyn, NY USA; 2 Department of Medicine, State University of New York Downstate Medical Center, Brooklyn, NY USA; 3 Department of Cell Biology, State University of New York Downstate Medical Center, Brooklyn, NY USA

**Keywords:** Immunology, Diseases, Medical research

## Abstract

Chronic rhinosinusitis (CRS) is a common condition associated with inflammation and tissue remodeling of the nose and paranasal sinuses, frequently occurring with nasal polyps and allergies. Here we investigate inflammation and the protease profile in nasal tissues and plasma from control non-CRS patients and CRS patients. Gene expression for several cytokines, proteases, and antiproteases was quantified in nasal tissue from non-CRS and CRS subjects with nasal polyps. Elevated expression of S100A9, IL1A, MMP3, MMP7, MMP11, MMP25, MMP28, and CTSK was observed in tissue from CRS subjects with nasal polyps compared to control tissue. Tissue protein analysis confirmed elevated levels of these targets compared to controls, and increased MMP3 and MMP7 observed in CRS subjects with nasal polyps compared to CRS subjects without polyps. Plasma concentrations of MMP3 and MMP7 were elevated in the CRS groups compared to controls. The nasal cell line, CCL-30, was exposed to S100A9 protein, resulting in increased MMP3, MMP7, and CTSK gene expression and elevated proliferation. Silencing MMP3 significantly reduced S100A9-mediated cell proliferation. Therefore, the elevated expression of S100A9 and MMPs are observed in CRS nasal tissue and S100A9 stimulated MMP3 responses to contribute to elevated nasal cell proliferation.

## Introduction

Sinusitis, often termed rhinosinusitis, is a condition associated with inflammation in the paranasal sinuses and contiguous nasal mucosa and nearly 30 million adults report sinusitis symptoms annually in the United States^1^. Sinusitis is classified according to the duration of symptoms as acute (< 1 month), subacute (1–3 months) or chronic (lasting more than 3 months). Most episodes of sinusitis are due to viral upper respiratory tract infections, that are linked to asthma, allergic rhinitis, and exposure to environmental factors, such as cigarette smoke^2,3^. The diagnosis of sinusitis is clinically determined, based on subjective and objective findings. Steroids are effective in treating sinusitis, thereby underlining the importance of inflammation cells and responses in disease pathophysiology^4^. Tissue remodeling occurs in CRS, and this remodeling is believed to be dependent on inflammation-mediated protease responses^5^. Nasal and sinus inflammation are well studied in CRS^6,7^ with several investigators reporting elevated circulating systemic inflammation in CRS populations^8,9^. Equally, protease levels are elevated in many diseases and several proteases and their antiproteases are elevated in CRS, including, MMP-2, MMP-7, MMP-9, and TIMP-1^10^. However, a broader investigation into a wider range of tissue-specific proteases and circulating levels of proteases in CRS are warranted.

Here, we investigate the expression of multiple cytokine and proteases in control and CRS nasal tissue. Since inflammation and tissue remodeling are primary contributing factors in chronic rhinosinusitis, we expected to observe significant inflammation and protease changes locally in the nasal tissue that could also be observed in plasma. Utilizing patient samples and a nasal epithelial cell line, we found a unique S100A9-mediated protease profile in CRS samples that may contribute to the cellular proliferation in CRS. S100A9 is a member of the S100 proteins family, which are low molecular-weight proteins that serve diverse functions in a wide variety of cell types and tissues. Dysregulated expression of S100A9 was previously reported in CRS samples^11,12^. Overall, the findings from this study profiled local and systemic inflammation and protease changes in CRS patients and explore the role of S100A9 and its mediated proteases in proliferation.

## Results

### Patient demographics

Twenty-five patients with documented CRS (12 males and 13 females) and 25 non-CRS patients (12 males and 13 females) were prospectively recruited in the study (see Table 1). Twelve patients exhibited the polyposis phenotype (CRSwNP) and thirteen without nasal polyps (CRSsNP). The mean age, smoking history, comorbidity index, asthma, COPD, and gastroesophageal reflux disease (GERD) frequency was similar between the groups (Table 1). Tissue eosinophils were noted in fifteen of the CRS groups, compared to zero of the controls (p < 0.001). Six patients had a history of prior sinonasal surgery related to their CRS. Nasal tissue was only collected from five control patients.

**Table 1:** Demographic and disease characteristics of control and CRS patients.

	Control	CRSsNP	CRSwNP	P-value
Patients (n)	25	13	12	
Age (mean ± SD)	47.0 ± 3.1	43.0 ± 4.8	41.8 ± 3.9	0.544
Sex (M: F)	12:13	6/7	6/6	0.849
Smokers, n (%)	8 (32%)	3 (23%)	3 (25%)	0.462
Comorbidities (mean ± S.E.M.)	2.0 ± 0.3	1.6 ± 0.4	1.3 ± 0.4	0.375
Asthmatics, n (%)	5 (20%)	4 (30.8%)	3 (25%)	0.666
GERD, n (%)	3 (12%)	2 (15.4%)	1 (8.3%)	0.625
COPD, n (%)	2 (8%)	1 (7.7%)	1 (8.3%)	0.889
Tissue eosinophil positive, n (%)	0 (0%)	6 (46.2%)	9 (75%)	< 0.001

Data are presented as number (percentage) or mean ± SD. Data were analyzed by D'Agostino & Pearson omnibus normality test and further analyzed by Student t-tests.

### Proteases are dysregulated in nasal tissue of CRS patients

Several cytokines and proteases were examined in nasal tissue from five non-CRS controls and five CRSwNP subjects (see Table 2 for patient demographics for PCR analysis). Of the 32 cytokines and chemokines examined, only *S100A9* and *IL1A* were significantly altered in samples from CRSwNP compared to controls (Table 3 and Supplemental Table S1). The expression of multiple proteases was elevated in tissue from CRS subjects (Table 3). There was significantly increased fold expression of *MMP3* (P = 0.005), *MMP7* (P = 0.032), *MMP11* (P = 0.003), *MMP25* (P < 0.001), *MMP28* (P = 0.024) and *CTSK* (P = 0.008). There were no changes in expression for the remainder of the MMPs, tissue inhibitors of MMPs (TIMP), cathepsins (CTS), or cystatins (CST) (Supplemental Table S2).

**Table 2:** Demographic and disease characteristics of control and CRSwNP patients for PCR analysis.

	Control	CRSwNP	P-value
Patients (n)	5	5	
Age (mean ± SD)	44.8 ± 12.4	42.0 ± 17.7	0.780
Sex (M: F)	3:2	2/3	0.580
Smokers, n (%)	0 (0%)	0 (0%)	
Comorbidities (mean ± S.E.M.)	1.2 ± 1.1	1.6 ± 1.5	0.913
Asthmatics, n (%)	0 (0%)	1 (25%)	
GERD, n (%)	0 (0%)	1 (20%)	
COPD, n (%)	0 (0%)	0 (0%)	
Tissue eosinophil positive, n (%)	0 (0%)	4 (80%)	

Data are presented as number (percentage) or mean ± SD. Data were analyzed by D'Agostino & Pearson omnibus normality test and further analyzed by Student t-tests.

**Table 3:** Nasal tissue gene expressions in CRSwNP compared to controls.

Gene	Fold change	P-value
*S100A9*	4.38 ± 1.2	** < 0.001**
*IL1A*	5.4 ± 1.3	**0.015**
*MMP3*	23.94 ± 2.9	**0.005**
*MMP7*	3.17 ± 1.1	**0.032**
*MMP11*	2.69 ± 0.5	**0.003**
*MMP25*	3.05 ± 0.7	** < 0.001**
*MMP28*	1.95 ± 0.5	**0.024**
*CTSK*	0.66 ± 0.3	**0.008**

Data is represented as gene expression fold change (+ /- SD) compared to control group, where n = 5 subjects per group. Data were analyzed by D'Agostino & Pearson omnibus normality test and further analyzed by Student’s t-test (two-tailed). P-values are shown here and significant differences in bold.

**Figure 1:**
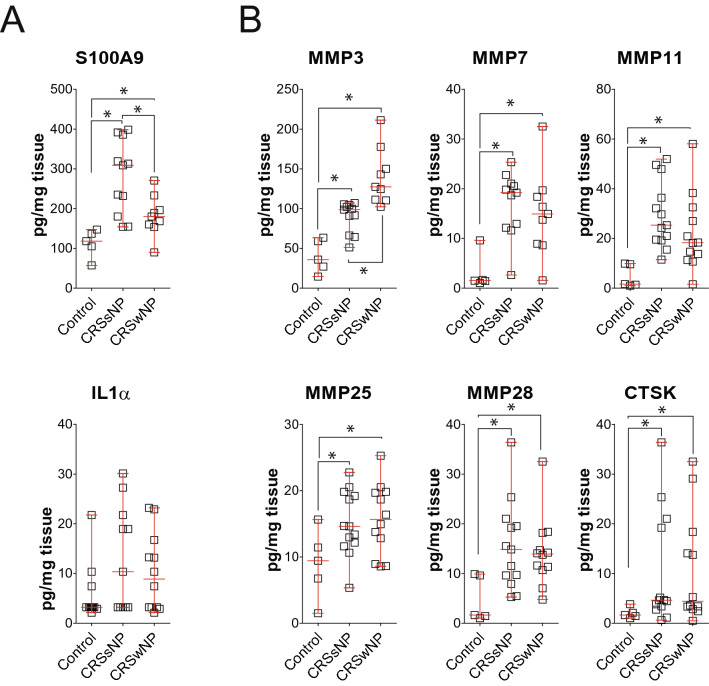
Elevated S100A9 and MMP protein levels in CRS nasal tissues. ELISAs were performed for (**A**) S100A9, IL1α, (**B**) MMP3, 7, 11, 28 and CTSK from protein isolated from non-CRS controls, CRSwNP and CRSsNP nasal tissues. Data is represented as pg/mg tissue protein, as mean ± SD. Data were analyzed by D'Agostino & Pearson omnibus normality test and further analyzed by the Mann Whitney test. * denotes a P-Value < 0.05 between the 2 groups connected by a line.

ELISA or Luminex assays were performed on protein samples from nasal tissue isolated from controls, CRSsNP, and CRSwNP subjects to confirm the PCR findings. Elevated S100A9 was confirmed in nasal tissue proteins (Fig. 1A). However, IL1α levels did not reach significance. Protein levels of MMP3, MMP7, MMP11, MMP25, MMP28, and CTSK were significantly elevated in CRSsNP and CRSwNP groups compared to controls (Fig. 1B). Elevated MMP3 was observed in the CRSwNP group compared to CRSsNP subjects.

### Plasma MMP3 and MMP7 differ in CRS patients compared to Non-CRS patients

**Figure 2:**
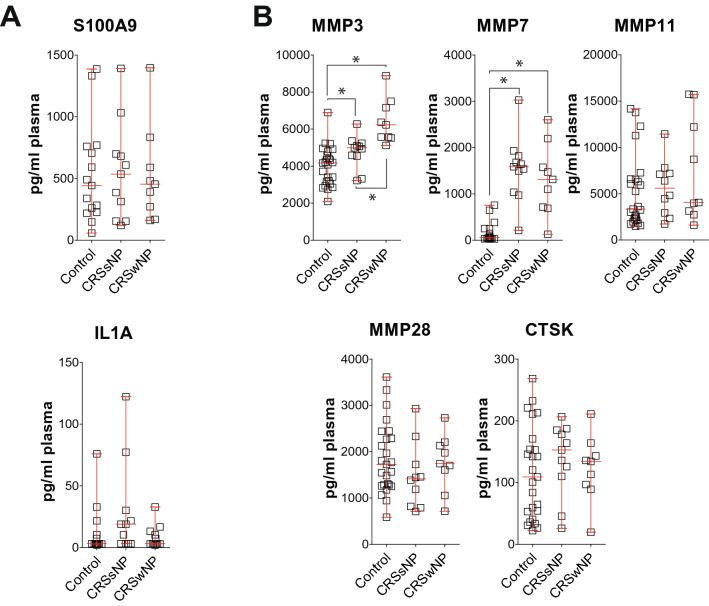
Elevated MMP3 and MMP7 levels in plasma from CRS subjects. Luminex bead assays and ELISAs were performed for (**A**) S100A9, IL1α, (**B**) MMP3, 7, 11, 28, and CTSK from plasma isolated from non-CRS controls, CRSwNP, and CRSsNP subjects. Data is represented as pg/ml plasma, as mean ± SD. Data were analyzed by D'Agostino & Pearson omnibus normality test and further analyzed by the Mann Whitney test. * denotes a P-Value < 0.05 between the 2 groups connected by a line.

Multiplex bead-based immunoassays and ELISAs were performed to measure the same targets in plasma samples from CRS patients and non-CRS controls. There were significantly higher concentrations of MMP3 and MMP7 observed in CRS plasma compared to controls (Fig. 2). The remainder of the targets tested were unchanged between groups. MMP-25 was not detectable. Similar to analysis in nasal tissue, elevated plasma MMP3 was observed in the CRSwNP group compared to CRSsNP subjects. Tissue and plasma levels of S100A9 and MMP3 were not influenced by the presence of tissue eosinophils (Supplementary Fig. 1).

### S100A9-induced MMP3 contributes to nasal epithelial cell proliferation

**Figure 3:**
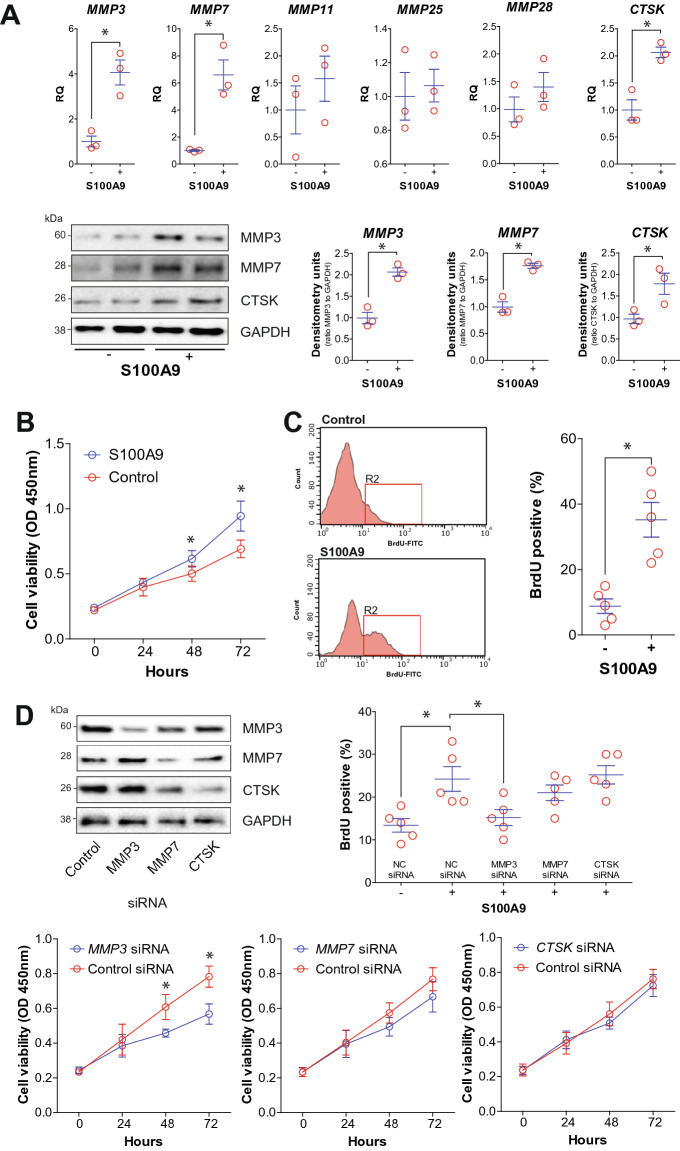
S100A9 triggers MMP3 induced nasal cell proliferation. (**A**) CCL-30 cells were exposed to 1 μg/ml S100A9 for 24 h. qPCR was performed for *MMP3*, *MMP7*, *MMP11*, *MMP25, MMP28*, and *CTSK*. Immunoblots and densitometry analysis confirmed our qPCR data for MMP3, MMP7 and CTSK. (**B**) The proliferation ability of S100A9 stimuli on CCL-30 cells was measured using the CCK8 assay and bromodeoxyuridine (BrdU) incorporation assays. (**D**) Gene expression was silenced for MMP3, MMP7, and CTSK in CCL-30 cells, and BrdU incorporation and CCK8 assays were performed. (**C**,**D**) BrdU positive cells were quantified 48 h after S100A9 stimuli. Data is represented as mean ± SD. Data were analyzed by D'Agostino & Pearson omnibus normality test and further analyzed by Student T-tests. * denotes a P-Value < 0.05 between the 2 groups connected by a line or at the same timepoint.

Since elevated S100A9 and proteases were observed in nasal tissue, we examined whether S100A9 mediates the expression of these proteases. Using the CCL-30 cell line, S100A9 stimuli was determined to induced the expression of *MMP3*, *MMP7*, and *CTSK* (Fig. 3A). Immunoblots confirmed elevated levels of MMP3, MMP7 and CTSK protein following S100A9 stimulation (Fig. 3A). Since S100A9 promotes human embryo lung fibroblast proliferation^13^, we examined whether S100A9 stimulation could promote nasal epithelial cell proliferation. Utilizing two assays, we determined that S100A9 stimulation-induced the proliferation of CCL-30 cells (Fig. 3B-C). Silencing gene expression of *MMP3* reduced S100A9-mediated proliferation, unlike *MMP7* or *CTSK* (Fig. 3D). Therefore, S100A9 signaling could contribute to MMP3, MMP7 and CTSK expression in nasal epithelium and MMP3 contribute to S100A9-induced proliferation.

## Discussion

This study sought to identify novel inflammatory and protease profiles in nasal tissues of CRS and non-CRS control subjects and determine the role of these targets in disease pathogenesis. Equally, we attempted to link changes in the nasal tissue with proliferation changes in nasal epithelium. The majority of the targets analyzed here in tissue were similar between control non-CRS patients and CRS patients. High expression levels of *S100A9*, *IL1A*, *MMP3*, *MMP7*, *MMP11*, *MMP25*, *MMP28*, and *CTSK*, were observed in the CRSwNP group compared to non-CRS controls. S100A9, MMP3, MMP7, MMP11, MMP25, MMP28, and CTSK protein levels were confirmed to be elevated in CRS nasal tissues. MMP3 levels were further elevated in CRS subjects with polyps. Both MMP3 and MMP7 were detected at higher concentrations in the plasma of CRS patients compared to controls. S100A9 stimuli induced nasal epithelial cell proliferation in an MMP3-dependent manner. These findings suggest that S100A9 could contribute to nasal protease expression and proliferation in CRS.

Currently, the CRS literature mainly lists elevated concentrations of MMP1, MMP8 and MMP13 (collagenases), MMP7 (multiple substrates), and MMP2, and MMP9 (both gelatinase)^10,14^ in CRS tissues. However, we have identified several other elevated proteases, such as MMP3, MMP11, MMP25, MMP28, and CTSK. Equally, we observed elevated plasma levels of MMP3 and MMP7. We are the first group to report this MMP3, MMP11, MMP25, and MMP28 changes in CRS. Currently, we know little about their functional role in CRS but will briefly discuss their possible roles here. Our nasal cell line data suggest that MMP3 plays an important role in cell proliferation and is S100A9 inducible. MMP3 is a protease that can degrade multiple components of the extracellular matrix^15^ and loss of MMP3 can prevent bleomycin-induced fibrosis in mice^16^. MMP3 polymorphisms are linked with cancer development in COPD patients^17^. Therefore, MMP3 could play a role in fibrosis and cell proliferation in CRS. However, further analysis is needed on the direct role of MMP3 in CRS and whether MMP3 could be utilized as a biomarker for disease severity or symptom outcomes. Others report that MMP-7 expression is significantly increased in samples from CRS patients^10^. MMP-7 cleaves a wide variety of extracellular matrix proteins, such as collagen IV, fibronectin, laminin, and tenascin-C^18–20^. MMP7 also cleaves TNF-α and other MMPs^21^. MMP7 promotes cell proliferation^22^ but we observe no significant role of MMP7 in nasal cell proliferation. The absence of MMP7 protects mice from LPS-induced intestinal permeability and lethality, possibly due to MMP7 activation of β-defensins^23^. MMP-11 promotes cancer development by inhibiting apoptosis, and by enhancing migration and invasion of cancer cells^24^. Recently, MMP25 was shown to regulate innate immune responses by modulating NFκB responses, primarily in leukocytes^25^. We previously reported that smoke inhalation and RSV infection could enhance MMP28 levels in mouse lungs^26^. Others have reported that MMP28 contributes to tumor cell invasion and metastasis^27^. There is little information on CTSK in CRS but CSTK was previously reported to be induced by smoke exposure^28^ and is overexpressed in many cancers including oral squamous cell carcinomas^29^. CTSK can catabolize elastin, collagen, and gelatin, which aids its breakdown of bone and cartilage^30^. Within our study, CTSK gene expression was lower in the CRSwNP group but higher when examining protein levels. S100A9 positively induced its expression. The majority of the CRS subjects had similar levels to controls but nine subjects had high levels of tissue CTSK. Sex, presence of polyps, smoking status, and eosinophilia did not influence these changes in our population. However, further studies are required to examine CTSK in CRS. Collectively, the functional role of each of these proteases in CRS requires investigation but the literature suggests that they play a role in extracellular matrix remodeling, immune response, and cell fate (apoptosis, proliferation, differentiation, and migration).

We observe elevated *S100A9* and *IL1A* gene expression in the nasal tissue of CRS subjects. There is an association between the IL1A polymorphism and severe CRS^31^. Interestingly, S100A8, S100A9, and S100A8/A9 protein levels were reported to be significantly higher in CRSwNP patients possibly due to upper airway infections^12^. The elevated S100A9 was more pronounced in the CRSsNP, which was a similar observation determined by another study^32^. Normally, S100A9 is expressed at low levels in healthy tissue but elevated levels are frequently observed in diseased tissue^33^, in addition to infiltrating immune cells^34^. Intracellularly, S100A9 is known to regulate NADPH oxidase activity^34^, which is a major source of reactive oxygen species in neutrophils. Extracellularly, high concentrations of S100A9 is observed at tissues with elevated inflammation or in the serum of patients with inflammatory diseases^35^. We previously reported that elevated S100A9 protein resulted in enhanced lung damage during smoke exposure and respiratory syncytial virus (RSV) infection^36^. It is plausible that S100A9 could stimulate proliferation and downstream remodeling via MMP-3 induction in the nasal tissue, as well as mediate tissue damage in CRS. S100A9 was not altered in peripheral blood specimens, which may be attributed to S100A9′s local manifestation of inflammatory activity^37^. S100A9 binds to EMMPRIN, an inducer of MMP synthesis, to regulate MMP1 expression in a melanoma cell model^38^. In human osteoarthritis synovium, paquinimod treatment blocked MMP1 and MMP3 secretion^39^. Here we observed that MMP3 is sensitive to S100A9 signaling and both contribute to nasal epithelial cell proliferation. Further studies are required to demonstrate whether S100A9 directly contributes to CRS progression and if treatments like paquinimod could be utilized in treating CRS.

Several limitations need to be discussed here. Specimens were predominantly from the middle turbinate in origin but, there were possibly some olfactory neuroepithelium present. This could influence the PCR profile we observed for several genes, as we observed no Th2 response. Our sample size is small and this could influence several outcomes. It would be beneficial to confirm our outcomes in a larger cohort of subjects. Finally, while S100A9 was elevated in CRSwNP compared to controls, the CRSsNP has the highest levels of S100A9 but the CRSwNP had the highest concentrations of MMP3. Therefore, additional factors must be contributing to MMP3 levels in CRS.

In conclusion, we profile several inflammation and protease responses in CRS patients and determine that elevated levels of S100A9 and proteases are observed in the nasal tissue of CRS subjects. Elevated levels of MMP3 in the nasal tissue is sensitive to S100A9 signaling and both contribute to nasal epithelial cell proliferation.

## Materials and methods

### Study population

Patients with documented chronic rhinosinusitis, who met diagnostic criteria set forth by the Adult Sinusitis Clinical Practice Guideline^40^ were prospectively recruited from a subspecialty, referral-based rhinology practice affiliated with SUNY Downstate Medical Center from January 2018 to September 2018. Diagnosis of chronic rhinosinusitis was made by a senior rhinologist based on a combination of clinical history and objective findings of mucosal inflammation, purulence, or polyposis on either nasal endoscopy or radiographic imaging. Specifically, relevant clinical history entails one or more of the following symptoms: mucopurulent discharge, nasal obstruction, facial pain or pressure, or decreased sensation of smell. Both polyposis phenotype positive (CRSwNP) and negative (CRSsNP) patients were recruited.

All patients had previously failed maximal medical treatment for more than three months, including topical nasal saline sprays, inhaled corticosteroids, and oral antibiotics, to control their CRS. All patients received three to five days of preoperative oral corticosteroids (Prednisone 40 mg daily) before nasal tissue and blood collection on the recommendation of a senior rhinologist. Specimens were all collected from the middle turbinate with some olfactory neuroepithelium and was often polypoid. There was no inferior turbinate or uncinate or other various sinuses present in the specimen. All specimens were collected by the same rhinologist for consist sampling. Specimens were evaluated by a pathologist. Tissue eosinophilia was defined as > 10 eosinophils under high power field (× 400 magnification) when examined by the pathologist.

Control non-CRS patients (> 18 years old) were recruited from the general ambulatory clinics at the same institution during that time-period, given that they were adults who did not meet the predetermined definitions for CRS. Patients were only selected for this study if they had no history for the following exclusion criteria: sickle cell disease, pulmonary artery hypertension, recent transfusion of packed red blood cells, acute coronary syndrome, life-threatening bleeding, hypercapnia, hypothermia, severe acidosis and significant anemia hemoglobin < 7 g/dL. Written and informed consent was obtained from all study participants and the study was approved by the institutional review board of the State University of New York Downstate Medical Center. All methods were performed in accordance with the institutional review board guidelines and regulations.

### Gene expression

Nasal tissue was snap-frozen and stored until RNA isolation. RNA was isolated using the Zymo Research kit following tissue homogenizing by bead beater disruption (Minibeadbeater-16, BioSpec Products, Bartlesville, OK, USA) and cDNA was reverse transcribed using the Applied Biosystems high capacity cDNA kit. qPCR was performed on the Bio-Rad CFX384 real-time system. No cDNA template, no reverse transcriptase treated samples, and no DNA polymerase controls were examined for each qPCR throughout this study. Exogenous (human targets) and endogenous (*βACT* and *GAPDH*) positive controls were also monitored for each assay. Gene expression was performed by qPCR using Taqman probes (Life Technologies/Applied Biosystems, Carlsbad, CA, USA). Data were analyzed using the ΔΔCT method, with normalization to β-actin and GAPDH, and are presented fold change compared to non-CRS controls.

### Cytokine and protease detection

Peripheral blood was collected and plasma was isolated and stored at − 80 °C until assays were performed. Plasma was defrosted and centrifuged minutes before the assay. All samples were analyzed for IL-1α (Millipore Chemokine and Cytokine Assay, Millipore), MMP-3, and -7 were investigated with magnetic bead Luminex assays (R&D Systems). S100A9 (R&D Systems), MMP11, MMP25, MMP28 (mybiosource.com), and CTSK (Novus Biologicals) were measured by ELISA. Each sample was run in triplicate. The coefficient of variation (CV) was performed for triplicate wells and CV > 20% were removed.

### Proliferation assays

The RPMI 2650/CCL-30 (ATCC) nasal epithelium cell line was cultured in Eagle's Minimum Essential Medium supplemented with 10% fetal calf serum. Cells were incubated in a humidified incubator at 36 °C in an atmosphere containing 5% CO_2_. Cells were only used for experiments at passages 5–10. Cells were transfected by administering silencing RNA (siRNA) for MMP3, MMP7, CTSK, or control siRNA (Qiagen, Gaithersburg, MD). Cells were stimulated with 1 μg/ml S100A9 recombinant protein (Novus Biologicals).

Cell proliferation was measured by counting the cells in the logarithmic phase using Cell Counting Kit-8 (#CK04; Dojindo Kumamoto, Japan) as previously outlined^22^. The cells were first transfected with siRNA or plasmid and then were plated into a 96-well plate. Cells from each group were plated in 3 wells, and each well contained 2 × 10^3^ cells. The absorbance of each well was measured with a reader at the same time over 3 consecutive days. This process was repeated in triplicate for the statistical analyses. For confirmation, the BrdU proliferation assay (BD Biosciences, San Jose, CA, USA) was performed according to the manufacturer’s instructions. The stained cells were analyzed in on the Guava easyCyte flow cytometer from Millipore.

### Immunoblotting

Protein was collected from cells using lysis buffer (10 mM HEPES (pH 7.9), 1.5 mM MgCl_2_, 10 mM KCl, 0.5 mM PMSF, 0.5 mM DTT, 0.2% Igepal CA-630). Soluble proteins were collected following 10-min centrifugation at 13,000 × g at 4 °C. Immunoblots for MMP-3 (anti-MMP3 monoclonal rabbit antibody against a synthetic peptide corresponding to residues close to the Ser417 of human MMP-3 protein; Cat. # 14351S), MMP-7 (anti-MMP7 monoclonal rabbit antibody against a synthetic peptide corresponding to residues surrounding Ile264 of mouse MMP-7 protein; Cat. # 3801S), CTSK (anti-CTSK monoclonal rabbit antibody against a synthetic peptide corresponding to a region within amino acids 300–329 of Rat Cathepsin K which includes residue 321; Cat. # ab19027, Abcam), and GAPDH (anti-GAPDH monoclonal rabbit antibody against a synthetic peptide corresponding to residues near the carboxy terminus of human GAPDH; Cat. # 5174S) (unless specified all antibodies from Cell Signaling Technologies, Beverly, MA, USA). Chemiluminescence detection was performed using the Bio-Rad Laboratories Molecular Imager ChemiDoc XRS + imaging system. Uncropped immunoblots are present in supplementary Fig. 2.

### Statistics

All analysis was performed using GraphPad Prism Software (Version 6 for Mac OS X). Demographics were interpreted using descriptive statistics. Normality testing (D'Agostino & Pearson omnibus normality test) was performed on all data sets. A comparison of groups was performed by Student’s t-test (two-tailed) when data passed the normality test and by Mann Whitney test if data did not pass normality testing. A P-value < 0.05 was regarded as statistically significant.

## Supplementary information

Supplementary information.
