# Supplementary material for: Elevated S100A9 expression in chronic rhinosinusitis coincides with elevated MMP production and proliferation in vitro
Source: Sci Rep. 2020 Oct 1;10:16350. doi: 10.1038/s41598-020-73480-8 (PMC7530678; doi:10.1038/s41598-020-73480-8)
Supplement: Supplementary file 1 — Supplementary information. [file 41598_2020_73480_MOESM1_ESM.pdf]

Elevated S100A9 expression in Chronic Rhinosinusitis coincides with elevated MMP production and proliferation *in vitro*

Marina Boruk[1#], Christopher Railwah[2#], Alnardo Lora[2], Sridesh Nath[2], Derek Wu[1], Lillian Chow[2], Panid Borhanjoo[2], Abdoulaye J. Dabo[2,3], Sadakat Chowdhury[2], Ryan Kaiser[2], Robert F. Foronjy[2,3], Richard Rosenfeld[1], and Patrick Geraghty[2,3,*]

[#]Both authors contributed equally

[1]Department of Otolaryngology, State University of New York Downstate Medical Center, Brooklyn, NY, USA; [2]Department of Medicine, State University of New York Downstate Medical Center, Brooklyn, NY, USA; [3]Department of Cell Biology, State University of New York Downstate Medical Center, Brooklyn, NY, USA

*Correspondence: Patrick.Geraghty@downstate.edu; Tel.: +01 718 270 3141 (P.G.)

Supplementary Material

Supplemental Table S1. Nasal tissue cytokine, chemokine and growth factor gene expressions

Gene	Fold Change	P-value
CCL11	2.91±0.7	0.515
CSF3	1.9±0.2	0.342
IFNA2	1.5±0.6	0.159
IFNG	2.9±0.8	0.508
IL10	2.4±0.4	0.077
IL12A	1.6±0.4	0.434
IL12B	2.6±0.3	0.073
IL13	1.3±0.5	0.835
IL15	1.3±0.6	0.522
IL17A	1.8±0.7	0.111
IL1RAP	1.7±0.6	0.175
IL1B	1.6±0.5	0.057
IL2	1.5±0.1	0.159
IL3	ND	-
IL4	0.6±0.1	0.477
IL5	2.1±0.9	0.638
IL6	1.8±0.6	0.517
IL7	3.1±0.8	0.086
CXCL8	4.7±0.9	0.052
CXCL10	3.6±0.5	0.216
CCL2	0.6±0.4	0.321
CCL3	1.1±0.3	0.826
CCL4	0.6±0.5	0.302
TNF	2.9±0.3	0.121
LTA	1.9±0.5	0.170
LIF	1.2±0.4	0.430
IL1RA	1.75±0.8	0.175
CXCL10	1.65±0.4	0.216
CCL2	0.68±0.3	0.543
VEGF	1.20±0.4	0.222

Data is represented as gene expression fold change (+/- SD) compared to control group, where n = 5 subjects per group. Data were analyzed by D'Agostino & Pearson omnibus normality test and further analyzed by Student’s t-test (two-tailed). P-values are shown here. ND denote not detectable.

Supplemental Table S2. Nasal tissue protease and antiprotease gene expressions

Gene	Fold Change	P-value	Gene	Fold Change	P-value
CTSE	1.2±0.3	0.130	*MMP1*	0.78±0.4	0.512
CTSF	1.3±0.5	0.385	*MMP2*	0.9±0.4	0.513
CTSG	0.8±0.4	0.676	*MMP8*	1.82±0.8	0.139
CTSH	0.9±0.2	0.545	*MMP9*	0.7±0.5	0.765
CTSL	0.7±0.4	0.058	*MMP10*	0.7±0.4	0.513
CTSO	1.1±0.3	0.420	*MMP12*	1.27±0.3	0.590
CTSS	1.02±0.4	0.921	*MMP13*	1.14±0.4	0.400
CTSV	1.1±0.6	0.741	*MMP14*	1.1±0.6	0.713
CTSW	0.9±0.4	0.872	*MMP15*	1.3±0.4	0.180
CTSZ	1.0±0.5	0.680	*MMP16*	1.1±0.6	0.705
CST1	3.0±0.9	0.054	*MMP17*	1.3±0.4	0.413
CST11	1.8±0.7	0.202	*MMP19*	1.1±0.7	0.781
CST13P	2.0±0.8	0.638	*MMP20*	1.8±0.4	0.202
CST2	1.7±0.4	0.421	*MMP21*	1.1±0.5	0.715
CST3	1.0±0.5	0.916	*MMP23A*	0.9±0.8	0.908
CST4	0.7±0.3	0.572	*MMP23B*	1.2±0.3	0.612
CST5	1.4±0.6	0.786	*MMP24*	1.0±0.3	0.455
CST6	3.4±0.8	0.320	*MMP26*	1.4±0.5	0.638
CST7	3.8±0.9	0.273	*MMP27*	1.9±0.4	0.371
CST8	1.8±0.5	0.202	*CTSA*	1.1±0.6	0.508
CST9	1.2±0.4	0.130	*CTSB*	0.9±0.2	0.661
CST9L	0.9±0.5	0.959	*CTSC*	0.8±0.3	0.360
CSTA	2.6±0.9	0.182	*CTSD*	1.0±0.5	0.705
CSTB	1.6±0.7	0.077	*TIMP1*	0.7±0.3	0.198
CSTF1	0.9±0.3	0.808	*TIMP2*	1.1±0.5	0.530
CSTF2	0.8±0.4	0.592	*TIMP3*	0.8±0.4	0.471
CSTF3	0.8±0.5	0.501	*TIMP4*	1.1±0.6	0.890
CSTL1	2.2±0.8	0.203			

Data is represented as gene expression fold change (+/- SD) compared to control group, where n = 5 subjects per group. Data were analyzed by D'Agostino & Pearson omnibus normality test and further analyzed by Student's t-test (two-tailed). P-values are shown here.

Supplemental Figure 1

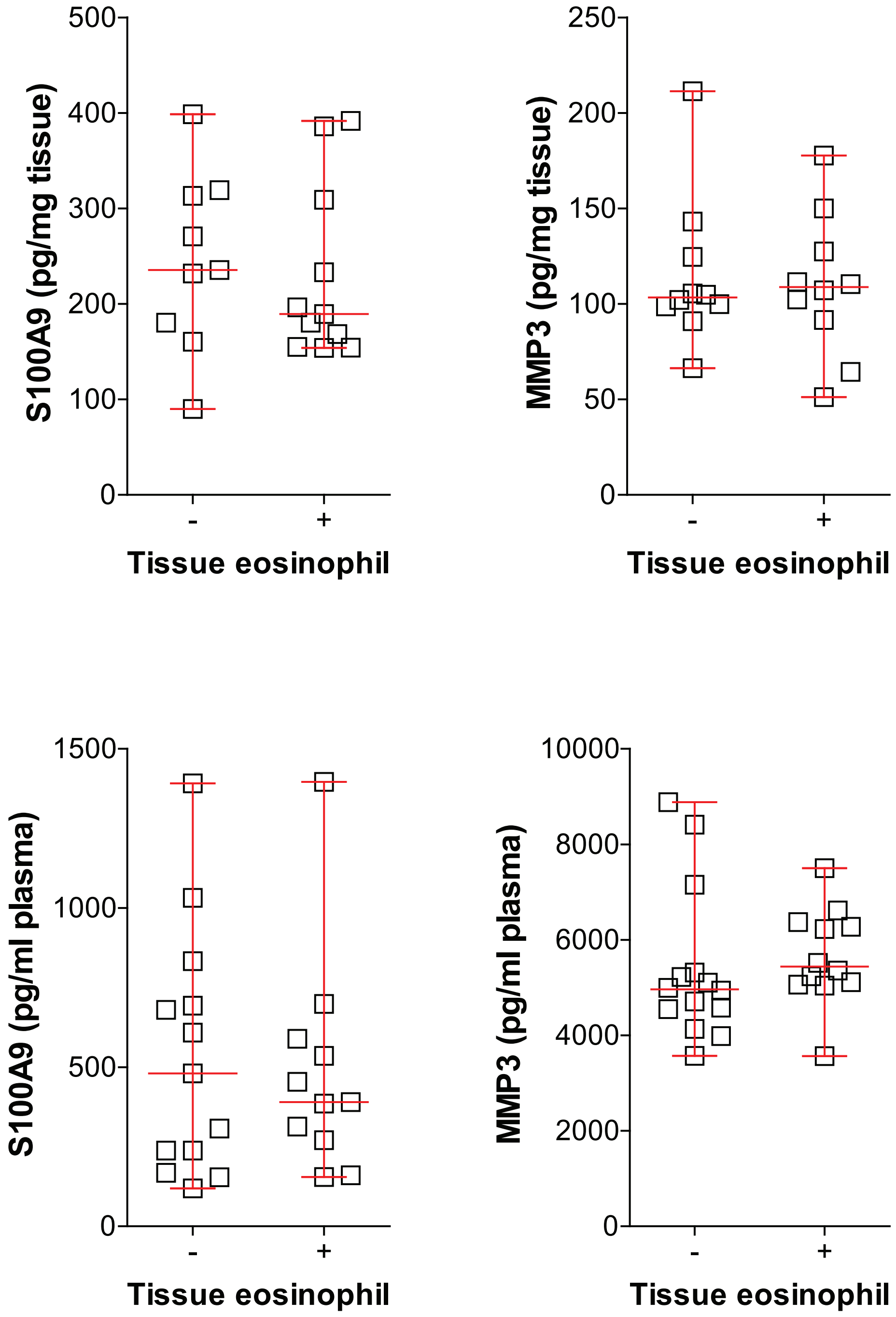

Supplemental Figure 1. S100A9 and MMP3 protein levels in CRS nasal tissues and plasma. ELISAs were performed for S100A9 and MMP3 from protein isolated from CRS with or without tissue eosinophils. Data is represented as pg/mg tissue protein, as mean ± SD. Data were analyzed by D'Agostino & Pearson omnibus normality test and further analyzed by the Mann Whitney test.

Supplementary Figure 2

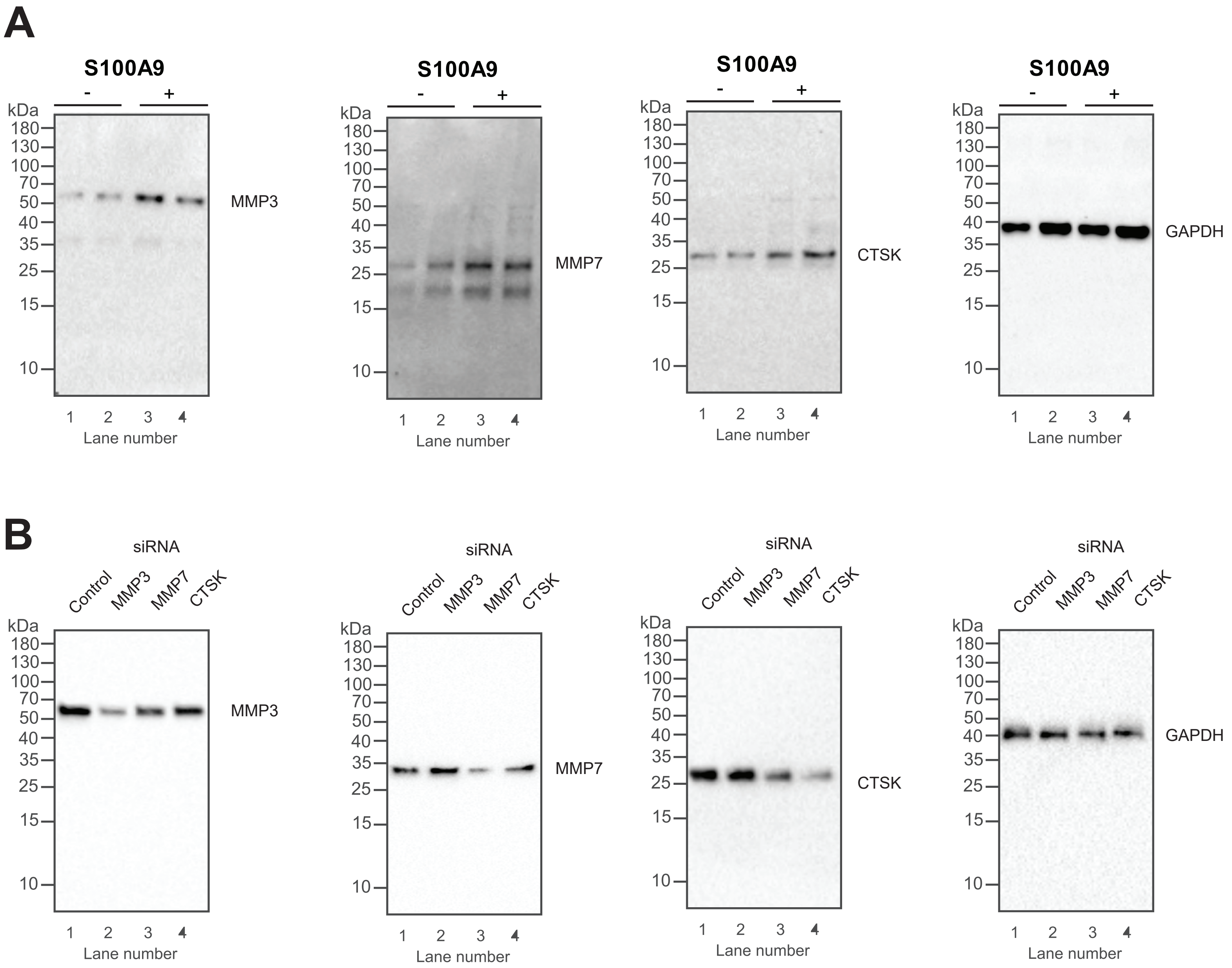

Supplemental Figure 2. Non-cropped pictures of blots. Non-cropped picture of immunoblots presented in Figure 3A (A) and 3D (B).
